# Assessment of Adherence to Consolidated Standards of Reporting Trials 2010 Guidelines of Randomized Controlled Trials Published in an Indian and International Pharmacology Journal From 2019 to 2023

**DOI:** 10.7759/cureus.80450

**Published:** 2025-03-12

**Authors:** Jitendra H Hotwani, Pankaj U Mahadkar, Ankita A Rao

**Affiliations:** 1 Pharmacology, Topiwala National Medical College and B. Y. L. Nair Charitable Hospital, Mumbai, IND

**Keywords:** chi-square test, clinical research, consolidated standards of reporting trials, fisher’s exact test, reporting guidelines

## Abstract

Randomized controlled trials (RCTs) are considered the gold standard in clinical research, providing the highest level of evidence for the effectiveness of healthcare interventions. However, the validity and utility of RCTs depend on the quality of their design, conduct, and reporting. The purpose of this review was to assess the adherence of RCTs published in Indian and international pharmacology journals to the Consolidated Standards of Reporting Trials (CONSORT) statement.

RCTs published from 2019 to 2023 from one Indian and one international pharmacology journal were assessed using the CONSORT 2010 checklist, and the items were assigned as "present" or "absent." Data was analyzed using descriptive statistics, and chi-square and Fisher’s exact tests were used for categorical data.

A total of 61 articles were analyzed, out of which 31 and 30 articles belonged to international and Indian journals, respectively. RCTs published in international journals consistently showed higher adherence rates compared to Indian journals, with statistically significant differences for several checklist items, including trial design description (31, 100%), intervention details (31, 100%), and reporting of harms (27, 87%) (p < 0.05). While Indian journals performed better on points like additional analyses (19, 63.3%) and recruitment dates (23, 76.6%). Overall, the international journal demonstrated significantly higher overall adherence to CONSORT guidelines compared to the Indian journal (p < 0.05).

The international journal exhibited greater overall adherence than the Indian journal, with the difference being statistically significant (p < 0.05). The overall reporting was suboptimal. Adherence should be improved further, and the journals should ensure the compliance of authors and reviewers with the standard reporting guidelines.

## Introduction and background

Carefully designed clinical trials and observational studies are crucial for providing the necessary information that enables clinicians to modify or adjust treatments effectively. The primary mode of communication among practicing physicians is peer-reviewed publication. Hence, it is important that the quality of such publications be maximized [1]. Randomized controlled trials (RCTs) are considered the gold standard in clinical research, providing the highest level of evidence for the effectiveness of healthcare interventions [2-4]. However, the validity and utility of RCTs depend on the quality of their design, conduct, and reporting [5]. Inadequate reporting can lead to biased results, reducing the reliability of the findings and potentially leading to incorrect clinical decisions [6]. The Consolidated Standards of Reporting Trials (CONSORT) statement, updated in 2010, describes guidelines for transparent and thorough reporting of RCTs, aiming to strengthen the reliability and quality of published trial reports [5]. Adherence to CONSORT guidelines ensures comprehensive documentation of trial methodology, conduct, and outcomes, facilitating accurate interpretation and replication of research findings [2].

In 1996, to improve clinical trial reporting, the CONSORT statement was developed. Since its inception, there have been significant updates in 2001 and 2010 [3,5]. The CONSORT 2010 guidelines provide a checklist of 25 items that should be included in the report of an RCT, covering aspects such as title and abstract, introduction, methods, results, discussion, and other information [2,5,7]. Strict compliance with the checklist items enhances the clarity, completeness, and transparency of reporting. Precise descriptions, nonambiguity, or omission best serve the interests of all readers [5].

Despite the widespread endorsement of the CONSORT guidelines by medical journals, adherence to these guidelines remains suboptimal [6]. Previous studies have shown that the reporting quality of RCTs varies widely across different journals and countries [8]. Evidence suggests that reports of low-quality RCTs as compared with higher-quality ones overestimate the effectiveness of interventions by about 30% across a variety of healthcare conditions [2,5]. RCTs that do not follow CONSORT guidelines while reporting yield unreliable and unpredictable results [9]. Readers of scientific literature deserve to know that editors, reviewers, and authors have adopted processes that foster clarity and replication [10].

This review aims to assess the adherence of RCTs published in Indian and international journals from 2019 to 2023 to CONSORT 2010 guidelines. The comparison with international journals will provide insights into the global trends and potential areas for improvement in the reporting of RCTs. Furthermore, investigating CONSORT adherence in both Indian and international journals allows for a comparative analysis that can reveal disparities and similarities in reporting practices across different research landscapes.

This article was previously presented as a poster at the 2024 Swaasthik Medical Conference on October 25, 2024.

## Review

Materials and methods

Research Design, Setting, and Approval

A cross-sectional, observational research project was conducted in the Department of Pharmacology, Topiwala National Medical College and B. Y. L. Nair Charitable Hospital, Mumbai, India, for a period of nine months, starting from May 2024 to January 2025. As there is no involvement of human or animal subjects in our review, ethics committee approval was not sought.

Selection Criteria

We selected two PUBMED-indexed pharmacology journals with comparable high-impact factors using Thomson Reuters journal citation reports 2022 [11], which were 2.4 and 2.9 for one Indian and one international journal, i.e., *Indian Journal of Pharmacology* (IJP) and *Biomed Central Pharmacology and Toxicology* (BMC), respectively. RCTs published in both these journals from 2019 to 2023 were selected.

Inclusion Criteria

We included published articles in which the research design was described as random, randomly allocated, randomized, or randomization.

Exclusion Criteria

Non-RCT research designs (e.g., observational studies, editorials, systematic reviews and meta-analyses, case reports, and letters to editors), studies not published in English, and studies with insufficient data for assessment were excluded.

Data Evaluation

Two reviewers received training on assessing RCTs based on the CONSORT 2010 guidelines. They independently extracted data from all selected studies, and any discrepancies were resolved through in-depth discussion. The articles were reviewed by two authors (PUM and AAR) using the CONSORT 2010 checklist items and the individual items were marked as "present" or "absent." Assessment of adherence to individual checklist items as well as overall adherence to the CONSORT statement was done.

Statistical Analysis

Data for descriptive statistics were presented using frequencies and percentages. Data was analyzed using Microsoft Excel 2021 (Microsoft Corp., Redmond, WA, USA) and GraphPad Prism 9 software (GraphPad Software, San Diego, CA, USA). Normality was tested using the Kolmogorov-Smirnov test, and statistical tests such as the chi-square test or Fisher’s exact test were used for categorical data. Statistical tests were summarized with a p-value <0.05 level of significance and a confidence interval of 95%.

Results

**Figure 1:**
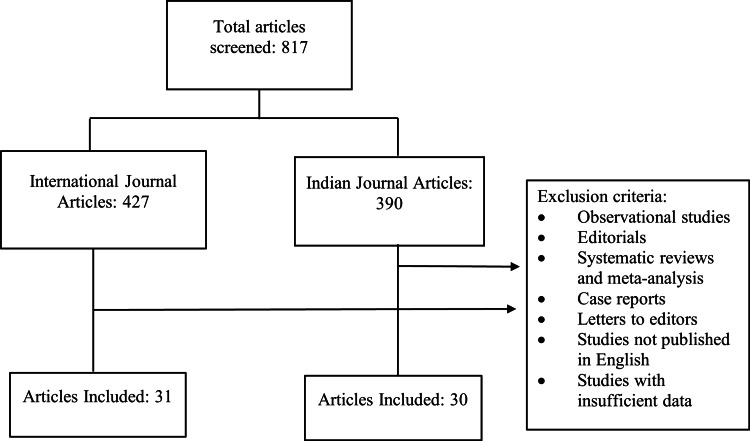
Study flowchart

Out of 817 articles screened in both journals from 2019 to 2023, a total of 61 articles were analyzed in our research, out of which 31 belonged to international and 30 belonged to Indian journals. Out of 427 articles published in international journals, 31 (7.25%) articles were RCTs, and out of 390 articles published in Indian journals, 30 (7.69%) articles were RCTs (Figure 1).

Adherence of International Journal Articles to Individual Items in the CONSORT 2010 Checklist

Analysis of articles in international journals to CONSORT guidelines uncovered varying levels of adherence. A full adherence rate, i.e., 31 (100%), was noted with 10 key checklist items: structured summaries of trial design, methods, results, and conclusions; specific objectives or hypotheses; descriptions of trial design; eligibility criteria for participants; details of interventions for each group; numbers of participants for each group; outcome results; their interpretation, benefits, and harms; funding; and trial registration.

However, our analysis uncovered some significant shortcomings. Less than 50% of the studies adhered to CONSORT guidelines with respect to the changes to methods after trial commencement with justifications - 5 (16%), prespecified primary and secondary outcome measures and their assessment methods - 7 (22.5%), reporting changes to trial outcomes after commencement - 3 (9.6%), interim analysis and stopping guidelines - 2 (6.5%), allocation concealment mechanism and its implementation - 15 (48.4%), blinding - 14 (45.1%), methods for additional analyses - 7 (22.5%), recruitment details - 11 (35.5%), absolute and relative effect sizes for binary outcomes - 2 (6.4%), results of any other analyses and accessibility to full trial protocols - 2 (6.5%).

**Figure 2:**
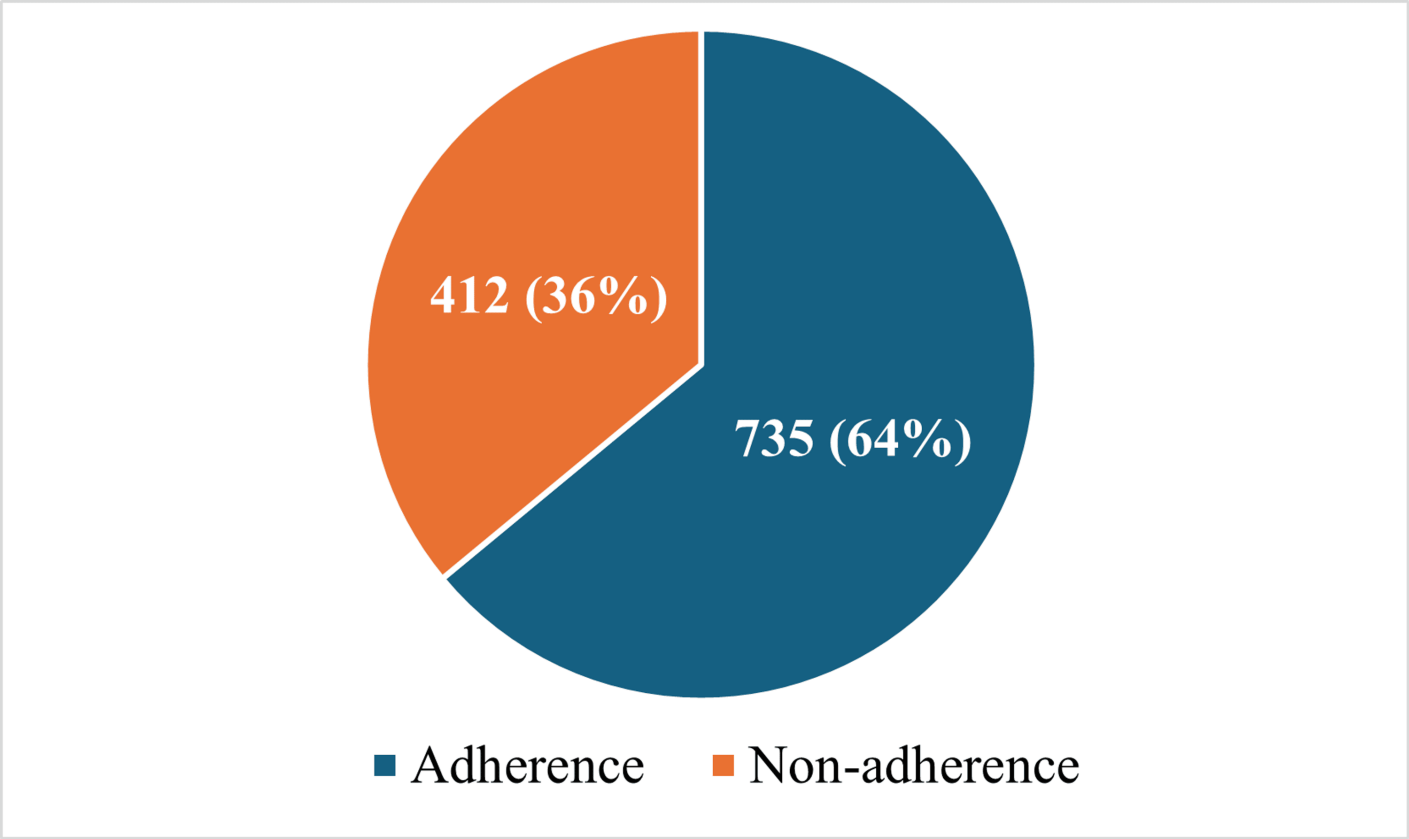
Adherence of articles in international journal

The overall adherence of the international journal to the CONSORT 2010 checklist was about 64% as demonstrated in Figure 2.

Adherence of Indian Journal Articles to Individual Items in the CONSORT 2010 Checklist

The articles adhered fully, i.e., 30 (100%), with respect to six checklist items: scientific background and explanation of rationale, specific objectives or hypotheses, eligibility criteria for participants, generalizability of findings, interpretation of results, and funding source.

Significant gaps were identified in several areas, with no adherence 0 (0%) observed for important changes to methods after trial commencement, any changes to trial outcomes after commencement, explanation of any interim analysis and stopping guidelines, description of similarity of interventions, reason for stopping of trial, for binary outcomes (absolute and relative effect sizes), and access to full protocol.

**Figure 3:**
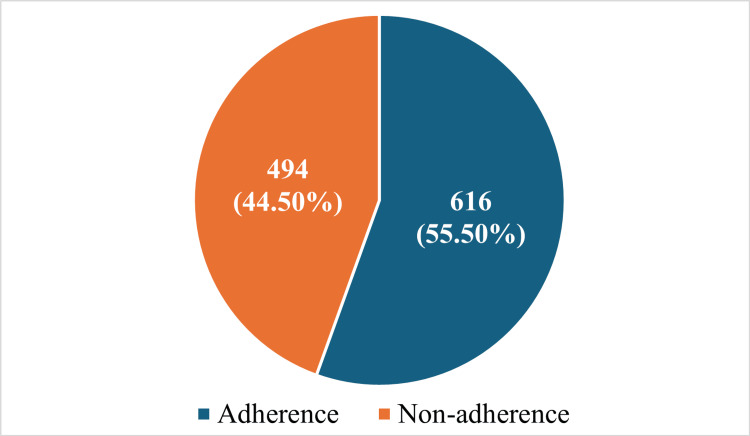
Adherence of articles in Indian journal

The overall adherence of the Indian journal to the CONSORT 2010 checklist was about 55.5% as demonstrated in Figure 3. The details of adherence of all the articles of both journals to the individual checklist items in CONSORT guidelines are given in Table 1.

**Table 1:** Adherence of all articles in BMC and IJP journals to the CONSORT 2010 guidelines BMC: *Biomed Central Pharmacology and Toxicology*; IJP: *Indian Journal of Pharmacology*; CONSORT: Consolidated Standards of Reporting Trials *p-value < 0.05 is considered statistically significant

		BMC journal (international journal) n = 31		IJP* *journal (Indian journal) n = 30		Calculated using chi-square test and Fisher’s exact test
Checklist items		Item present	Item absent	Item present	Item absent	p-value < 0.05 is significant
1a	Identification as a randomized trial in the title	19 (61.3%)	12 (38.7%)	21 (70%)	9 (30%)	p > 0.05
1b	Structured summary of trial design, methods, results, and conclusions (for specific guidance see CONSORT for abstracts)	31 (100%)	0 (0%)	29 (96.6%)	1 (3.4%)	p > 0.05
2a	Scientific background and explanation of rationale	27 (87%)	4 (13%)	30 (100%)	0 (0%)	p > 0.05
2b	Specific objectives or hypotheses	31 (100%)	0 (0%)	30 (100%)	0 (0%)	p > 0.05
3a	Description of trial design (such as parallel, factorial) including allocation ratio	31 (100%)	0 (0%)	23 (76.6%)	7 (23.3%)	p < 0.05*
3b	Important changes to methods after trial commencement (such as eligibility criteria), with reasons	5 (16%)	26 (84%)	0 (0%)	30 (100%)	p > 0.05
4a	Eligibility criteria for participants	31 (100%)	0 (0%)	30 (100%)	0 (0%)	p > 0.05
4b	Settings and locations where the data were collected	25 (80.6%)	6 (19.4%)	28 (93.3%)	2 (6.7%)	p > 0.05
5	The interventions for each group with sufficient details to allow replication, including how and when they were actually administered	31 (100%)	0 (0%)	25 (83.3%)	5 (16.7%)	p < 0.05*
6a	Completely defined pre-specified primary and secondary outcome measures, including how and when they were assessed	7 (22.5%)	24 (77.5%)	8 (26.6%)	22 (73.4%)	p > 0.05
6b	Any changes to trial outcomes after the trial commenced, with reasons	3 (9.6%)	28 (90.4%)	0 (0%)	30 (100%)	p > 0.05
7a	How sample size was determined	20 (64.5%)	11 (35.5%)	13 (43.4%)	17 (56.7%)	p > 0.05
7b	When applicable, explanation of any interim analyses and stopping guidelines	2 (6.5%)	29 (93.5%)	0 (0%)	30 (100%)	p > 0.05
8a	Method used to generate the random allocation sequence	22 (70.9%)	9 (29.1%)	23 (76.7%)	7 (23.3%)	p > 0.05
8b	Type of randomization; details of any restriction (such as blocking and block size)	24 (77.4%)	7 (22.6%)	17 (56.7%)	13 (43.4%)	p > 0.05
9	Mechanism used to implement the random allocation sequence (such as sequentially numbered containers), describing any steps taken to conceal the sequence until interventions were assigned	15 (48.4%)	16 (51.6%)	12 (40%)	18 (60%)	p > 0.05
10	Who generated the random allocation sequence, who enrolled participants, and who assigned participants to interventions	11 (35.5%)	20 (64.5%)	7 (23.3%)	23 (76.7%)	p > 0.05
11a	If done, who was blinded after assignment to interventions (for example, participants, care providers, those assessing outcomes) and how	14 (45.1%)	17 (54.9%)	11 (36.7%)	19 (63.3%)	p > 0.05
11b	If relevant, description of the similarity of interventions	15 (48.4%)	16 (51.6%)	0 (0%)	30 (100%)	p < 0.05*
12a	Statistical methods used to compare groups for primary and secondary outcomes	28 (90.3%)	3 (9.7%)	3 (10%)	27 (90%)	p < 0.05*
12b	Methods for additional analyses, such as subgroup analyses and adjusted analyses	7 (22.5%)	24 (22.5%)	19 (63.3%)	11 (36.7%)	p < 0.05*
13a	For each group, the numbers of participants who were randomly assigned, received intended treatment, and were analyzed for the primary outcome	30 (96.7%)	1 (3.3%)	19 (63.3%)	11 (36.7%)	p < 0.05*
13b	For each group, losses and exclusions after randomization, together with reasons	20 (64.5%)	11 (35.5%)	20 (66.7%)	10 (33.4%)	p > 0.05
14a	Dates defining the periods of recruitment and follow-up	11 (35.5%)	20 (65.5%)	23 (76.6%)	7 (23.3%)	p < 0.05*
14b	Why the trial ended or was stopped	3 (9.7%)	28 (90.3%)	0 (0%)	30 (100%)	p > 0.05
15	A table showing baseline demographic and clinical characteristics for each group	26 (83.9%)	5 (16.1%)	26 (86.7%)	4 (13.3%)	p > 0.05
16	For each group, number of participants (denominator) included in each analysis and whether the analysis was by original assigned groups	31 (100%)	0 (0%)	28 (93.3%)	2 (6.7%)	p > 0.05
17a	For each primary and secondary outcome, results for each group, and the estimated effect size and its precision (such as 95% confidence interval)	31 (100%)	0 (0%)	27 (90%)	3 (10%)	p > 0.05
17b	For binary outcomes, presentation of both absolute and relative effect sizes is recommended	2 (6.4%)	29 (93.6%)	0 (0%)	30 (100%)	p > 0.05
18	Results of any other analyses performed, including subgroup analyses and adjusted analyses, distinguishing pre-specified from exploratory	6 (19.4%)	25 (80.6%)	24 (80%)	6 (20%)	p < 0.05*
19	All-important harms or unintended effects in each group (for specific guidance see CONSORT for harms)	27 (87%)	4 (13%)	8 (26.7%)	22 (73.3%)	p < 0.05*
20	Trial limitations, addressing sources of potential bias, imprecision, and, if relevant, multiplicity of analyses	24 (77.5%)	7 (22.5%)	7 (23.3%)	23 (76.6%)	p < 0.05*
21	Generalizability (external validity, applicability) of the trial findings	30 (96.7%)	1 (3.3%)	30 (100%)	0 (0%)	p > 0.05
22	Interpretation consistent with results, balancing benefits and harms, and considering other relevant evidence	31 (100%)	0 (0%)	30 (100%)	0 (0%)	p > 0.05
23	Registration number and name of trial registry	31 (100%)	0 (0%)	15 (50%)	15 (50%)	p < 0.05*
24	Where the full trial protocol can be accessed, if available	2 (6.5%)	29 (93.5%)	0 (0%)	30 (100%)	p > 0.05
25	Sources of funding and other support (such as supply of drugs), role of funders	31 (100%)	0 (0%)	30 (100%)	0 (0%)	p > 0.05
Total		735 (64%)	412 (36%)	616 (55.5%)	494 (44.5%)	p < 0.05*

Comparison of Adherence Between the Two Journal Articles

**Figure 4:**
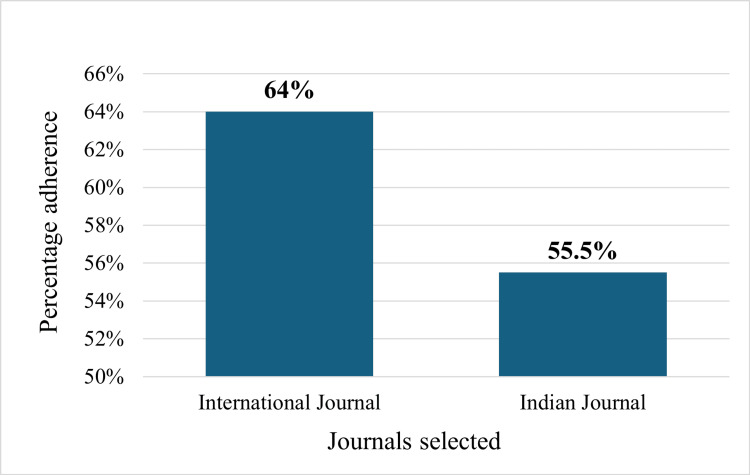
Overall adherence of international and Indian journal

In this review, we also compared the adherence between the two pharmacology journals chosen. A statistically significant difference (p < 0.05) was noted for some of the checklist items, such as items 3a, 5, 11b, 12a, 12b, 13a, 14a, 18, 19, 20, and 23. The details are mentioned in Table 1. Overall, a significant difference in adherence to CONSORT guidelines was observed between the two journals, with BMC journals demonstrating higher overall adherence (735, 64%) compared to IJP journals (616, 55.5%) (p < 0.05) as shown in Figure 4.

Discussion

By scrutinizing 61 articles, this review highlights significant discrepancies in adherence between the international and Indian pharmacology journals, which provide valuable insights into the current state of reporting quality in scientific publications.

The overall adherence of the IJP journal was 616 (55.5%), and that of the BMC journal was 735 (64%), which was a statistically significant difference (p < 0.05). The perfect adherence to item 3a (description of trial design including allocation ratio) in the BMC journal compared to the (23, 76.6%) adherence in the IJP journal underscores the rigorous reporting standards upheld by the BMC journal. This distinction is statistically significant (p < 0.05), suggesting that BMC journal may be more committed to ensuring clarity in trial design, which is crucial for reproducibility and understanding of the studies. Similarly, perfect adherence was also observed in the analysis conducted by Huang et al. on 182 RCTs [12]. The complete adherence of the BMC journal to item 5 (intervention details) versus the (25, 83.3%) adherence in the IJP journal further emphasizes the superior reporting quality in BMC publications, which was also observed in an analysis done by McErlean et al., which also showed 100% adherence to item 5 for the international journal [3]. This aspect is vital for the replication of studies and for clinicians to understand and apply the findings accurately. Details about pre-specified primary and secondary outcome measures (item 6a) had similar adherence by both the journals. This was also observed in studies done by Susvirkar et al. and Goenka et al. [4,13]. The disparity observed in item 11b (description of the similarity of interventions) - 15 (48.4%) in the BMC journal and 0 (0%) in the IJP journal - reveals a significant gap in reporting standards, which was also noted in studies by Sarveravan et al. and McErlean et al., reporting about 81% and 73%, respectively, for international journals [3,14].

The most likely cause of non-adherence to some items in IJP may be that the items themselves are not applicable to the articles, such as item 3b (important changes to methods after trial commencement), item 6b (changes to trial outcomes after the trial commencement), item 7b (explanation of interim analysis and stopping guidelines), and item 14b (why the trial ended or was stopped). This was similarly observed in the systematic reviews of 244 RCTs and 50 RCTs done by Singh et al. and McErlean et al., respectively [3,7].

Statistical methods for analysis should be described with enough detail to yield an estimate of the treatment effect, which is a contrast between the outcomes in the comparison groups [2]. Adherence to item 12a, which involves statistical methods used to compare groups for primary and secondary outcomes, was markedly higher in BMC journal articles, i.e., 28 (90.3%), than in IJP journal articles, 3 (10%). This observation was also corroborated by the studies conducted by Canagarajah et al. and Stevanovic et al., which yielded a result of approximately 95.1% and 98% for the international journal [15,16]. This suggests that the BMC journal may place a stronger emphasis on detailed statistical reporting for primary and secondary outcomes compared to the IJP journal. Conversely, adherence to item 12b, which refers to methods for additional analyses such as subgroup and adjusted analyses, was more frequently observed in IJP journal articles - 19 (63.3%) - compared to BMC journal articles - 7 (22.5%). This was found to be higher in our review as compared to the systematic review done by Singh et al. (16%) for the Indian journal [7]. This indicates that the IJP journal might be more focused on reporting additional analytical methods, potentially reflecting different editorial priorities or author practices.

Moreover, adherence to item 13a, which involves reporting the numbers of participants who were randomly assigned, received intended treatment, and were analyzed for the primary outcome, was significantly higher, amounting to 30 (96.7%) in BMC journal articles, which was also in accordance with the research done by Susvirkar et al. [4]. This disparity highlights a potential area for improvement in the reporting standards of the IJP journal, emphasizing the need for stricter adherence to CONSORT guidelines to enhance the transparency and reliability of published RCTs. Conversely, the IJP journal demonstrated better adherence to item 14a (dates defining recruitment and follow-up), which came to be 23 (76.6%), and item 18 (results of additional analyses), which was 24 (80%); this indicates that while the BMC journal excels in certain areas, the IJP journal may provide more comprehensive timelines and additional analysis results, which are also critical components of transparent and thorough reporting.

Sample size determination was reported more in BMC journals, though not adequately, as it is required to ensure that these publications are transparent and comprehensible [17]. According to the analysis done by Warrier and Jayanthi, out of 276 trials assessed, sample size determination of Indian journals during 2017, 2018, and 2019 was reported to be about 72%, 70%, and 77%, respectively, which was found to be higher as compared to our research, which was about 13 (43.4%) [18]. In the analysis of 25 RCTs done by Juneja et al., sample size determination was found to be 46%, which was in concordance with our analysis [19]. According to the analysis done by Susvirkar et al., sample size determination in international journals was found to be 97.9%, which was found to be higher as compared to our result of about 20 (64.5%) for the BMC journal [4].

Despite these strengths, the IJP journal showed significantly lower adherence in reporting important harms and unintended effects (item 19), with only 8 (26.7%) adherences as compared to BMC's 27 (87%). This discrepancy is concerning as it suggests a potential underreporting of adverse effects, which is crucial for assessing the safety profile of interventions.

Additionally, BMC journal's superior adherence, i.e., 24 (77.5%) to reporting trial limitations and addressing potential sources of bias versus IJP's 7 (23.3%) and their complete compliance with trial registration reporting accounting for 31 (100%) versus IJP’s 15 (50%) highlight areas where IJP journal needs substantial improvement.

Limitations

The focus on a specific time frame (2019-2023) may not capture long-term trends or improvements in adherence to CONSORT guidelines over different time periods. Instead of only two journals, multiple journals could have been analyzed to measure the wide variability and adherence of different journals to CONSORT 2010 guidelines. Lastly, we could not discuss adherence to all the checklist items due to the word constraint of the journal.

## Conclusions

This review highlights a significant variation in adherence to CONSORT 2010 guidelines between international and Indian pharmacology journals. The international journal demonstrated a higher overall adherence rate (64%) compared to the Indian journal (55.5%) (p < 0.05). Notably, the international journal excelled in reporting trial design, intervention details, harms, and statistical methods, whereas the Indian journal performed better in reporting additional analyses and recruitment details. However, both journals exhibited weaknesses in areas such as reporting changes to trial methodology, allocation concealment, and access to full trial protocols. To improve adherence, journals should enforce stricter editorial policies, provide reviewer training on guideline compliance, and encourage authors to follow best practices in trial reporting. Conducting workshops and establishing post-publication audits could further enhance adherence. Strengthening these measures will help ensure greater transparency, reproducibility, and reliability in the reporting of RCTs, ultimately improving the integrity of clinical research.
